# *Staphylococcus aureus* Infection in a Pediatric ICU: A Hospital Based Prospective Observational Study

**DOI:** 10.5005/jp-journals-10071-23162

**Published:** 2019-05

**Authors:** Ishtiyaq Qadri, Ishaq Malik, Kaisar Ahmed

**Affiliations:** 1-3 Department of Pediatrics, GB Pant Hospital, Srinagar, Jammu and Kashmir, India

**Keywords:** Mortality, MRSA, PICU, *Staphylococcus aureus*

## Abstract

**Introduction:**

Admission to a pediatric intensive care unit (PICU) with a *Staphylococcus aureus* (SA) infection is associated with considerable mortality and morbidity. There is paucity of data about epidemiology of SA infection in a PICU. This study was aimed at elucidating the clinicoepidemiological profile and outcome of children admitted to ICU with *S. aureus* infection.

**Methods:**

This study was carried out in a PICU at a tertiary care hospital in northern India. Children admitted with culture positive *S. aureus* infection were enrolled in this study. Children suspected of having *S. aureus* infection on clinical grounds only without a positive culture were excluded from the study. Baseline characteristics of the subjects were recorded on admission and daily follow up maintained till death or discharge from PICU. The course during PICU stay, ensuing complication, and outcome was recorded.

**Results:**

There were 2,480 total admissions to the PICU during study period of one year, out of which 120 (4.83%) admissions had a culture proven *S. aureus* infection. Fifty-six (46.6%) were male and 64 (53.3%) were female. Most of the subjects fell in the age groups of 1–5 years and 10–15 years having 56 (46.6%) and 40 (33.3%) subjects, respectively. Pneumonia (43.3%), septicaemia (20.8), and bone/joint space infections (15%) were the three main clinical manifestations. Forty-two (35%) of specimens were reported as methicillin resistant. Incidence of methicillin resistant SA (MRSA) infection was 1.6 and that of methicillin sensitive SA (MSSA) 3.1 per 100 admissions to PICU. On sensitivity testing, none of the specimens was found to be vancomycin resistant. There were 240 total deaths in PICU during study period out of which 25 (10.4%) were observed from the study group. Mortality rate was 20.8%. Mortality was high in the MRSA group.

**Conclusion:**

The incidence of *S. aureus* infection and associated mortality is high in PICU. MRSA infection was more common in children admitted with chronic disease and is associated with higher mortality. Our study found a bimodal age distribution for serious staph infection, a finding that needs further evaluation.

**How to cite this article:**

Qadri I, Malik I, Ahmed K. Staphylococcus aureus Infection in A Pediatric ICU: A Hospital Based Prospective Observational Study. Indian J Crit Care Med 2019;23(5):210-212.

## INTRODUCTION

*Staphylococcus aureus* is a leading cause of many serious bacterial infections with a wide range of clinical manifestations across all age groups with the highest rates of infection occurring at either extreme of life.^1^ Approximately 30% of the human population is colonized with *S. aureus* with a high percentage of colonization in healthy persons.^2^ It has an expanded armamentarium of virulence factors, which present it with unique survival and pathogenic properties. Over the years, there has been emergence of community-associated methicillin-resistant *S. aureus* (CA-MRSA) infections in immunocompetent individuals suggestive of an increased virulence of the bacterium possibly because of acquisition of novel genetic elements. There is abundance of literature on various aspects of staph infection in adults; however, data in children especially from a resource limited region is still scarce. Therefore, we tried to explore the epidemiological characteristics and outcome of staph infection in children in an ICU setting where a significant mortality due to this organism has been observed.

## METHODS

This was an observational prospective study conducted at the GB Pant Children Hospital, Srinagar, a tertiary care hospital for children from June 2016 to June 2017. A written informed consent was obtained from parents or guardians. Appropriate samples (blood, body fluids including C.S.F, and wound swabs) were taken from patients for culture on admission to PICU wherever indicated. Those patients beyond the neonatal age group admitted to the PICU with a culture proven *Staphylococcus aureus* infection were included in the study group. Children suspected of having SA infection on clinical grounds but with cultures negative for *S. aureus* were not included. Children found to have coagulase negative *S. aureus* infection on culture of their respective specimens were also excluded from the study. A detailed history was sought and thorough physical examination was done along with anthropometric measurements. Patients were monitored daily and their course in hospital, complications encountered, and outcome was recorded as per a pre-set proforma. Patients were managed as per standard guidelines.

## RESULTS

During the study period, there were a total of 2,480 admissions in PICU, out of which 120 met the inclusion criterion. Baseline characteristics of the study population are given in Table 1. Incidence of methicillin resistant SA (MRSA) and methicillin sensitive SA (MSSA) infection was 1.6 and 3.1, respectively per 100 admissions.

**Table 1:** Baseline characteristics

*Characteristic*	*N (%)*
Male	56 (46.6%)
Female	64 (53.3%)
Mean Age	5.6 years
Mean weight	21.6 kg
Mean height	118.3 cms
Underlying disorder	36 (30%)
Living below poverty line (as per KS scale)	13 (10.8%)
Malnutrion	20 (16.8%)
Hospital Acquired	38 (32.6%)
Community acquired	82 (68.3%)
MRSA	42 (35%)
MSSA	78 (65%)
Mean hospital stay	17 days

**Table 2:** Age distribution

*Age group*	*N (%)*
1–5 years	56 (46.6%)
5–10 years	15 (12.5%)
10–15 years	40 (33.3%)
>15 years	9 (7.5%)

**Table 3:** Clinical profile

Pneumonia	52 (43.35)
Septicaemia	24 (20%)
Bone/joint space infection	18 (15%)
Disseminated staph infection	10 (8.3%)
Skin/ soft tissue	9 (7.5%)
Meningitis	5 (4.1%)
Brain abscess	2 (1.6%)

**Table 4:** Major complications

*Complications*	*N*
Shock	32
Need for invasive ventilation	29
Respiratory failure	20
DIC	15
Empyema	10
Seizures	3
Raised ICT	1
Hemiparesis	1
ARF	1

Thirty-three patients out of 38 (86.8%) with a hospital acquired staph infection showed methicillin resistance and only five (13.1%) were due to MSSA. From 82 patients with community acquired infection, 73 (89%) cases were due to MSSA and only nine (10.9%) were due to MRSA. Our study found a bimodal age distribution of the disease with age groups 1–5 years and 10–15 years having bulk of the cases (80%) (Table 2).

The respiratory system was the major site of clinical presentation of *S. aureus* infection (43.3%) followed by septicaemia (20%) and bone/joint space infection (15%) as shown in Table 3.

Shock requiring vasopressors was the most frequent complication encountered. Respiratory failure requiring mechanical ventilation was the second most common complication followed by respiratory failure managed with non-invasive oxygen support and DIC (Table 4). Empyema was seen in ten patients. Complications were more frequent in the hospital acquired MRSA group. Mean hospital stay was 17 days with a maximum stay of 56 days and a minimum stay of 2 days.

During the study period, a total of 240 deaths occurred out of which 25/120 (10.5%) of total admissions took place among the study group. Mortality rate was 20.8% in the study group. Most of the deaths 20/25 (80%), however, occurred in those with an underlying chronic disease (like cerebral palsy, neuroregression, and cystic fibrosis). Most of deaths were recorded in the age group of 1–3 years (80%). Furthermore, a higher number of deaths 15/25 (72%) were recorded in the hospital acquired MRSA group.

## DISCUSSION

There is little data on Staphylococcal infection in children, in general. Our study is first at attempting to elaborate on clinicoepidemiological characteristics of staphylococcal infection in ICU admitted children.

The incidence of staph infection was 4.8 per 100 admissions to pediatric ICU which is higher than previous studies in children and adults.^3,4^ However these studies included all of hospital population while our study was conducted on an ICU based population, which might explain the resultant high incidence. Incidence of MRSA infection was 1.6 per 100 admissions to ICU, higher than a previous hospital based study from the USA.^5^ Again, this study calculated the incidence from the entire hospital cohort including neonates. Further, this difference could also be attributed to higher healthcare standards of a developed country. Many studies have found an increasing trend of hospital acquired MRSA infection.^6^ Our study also found a high incidence of hospital acquired MRSA infection. However, there is little substantial data on incidence of MRSA infection in children in the developing world with which we could compare our results.

Community acquired MRSA has been a growing problem as elucidated in many studies.^7^ In an Iranian study, a high frequency of MRSA was found not only in hospital acquired *S. aureus* but also in community acquired isolates.^8^ However in our study, MRSA infection was found more commonly as a hospital acquired infection. Also, it was particularly found to affect children having chronic disease or disability and was associated with greater mortality (72%). Studies done in adults have documented a high risk of mortality in patients with MRSA infection.^9^ MRSA infection was also associated with more complications. More virulent than ever, MRSA has the ability to become easily resistant every time a new therapeutic agent is introduced, except for vancomycin. Vancomycin has been considered useful for the past 40 years. However, increasing rates of treatment failure with strains are being reported.^10,11^ Mortality rate was 20.8 per 100 cases admitted with *S. aureus* infection. Further, maximum mortality was observed in the age group of 1–3 years (80%), which could be hypothesized by their relatively weaker immunity as compared to older children.

Our study found a bimodal age distribution of the disease with age groups 1–5 years and 10–15 years constituting most of the cases (80%). On review of literature, we did not find substantial data that could support or refute this observation. However, it has been our observation in our ICU that we do receive most cases of *S. aureus* from these two age groups. It may need further study to delve into this epidemiological observation further.

## CONCLUSION

Our study is the first to discuss *S. aureus* infection in a PICU. Staphylococcal infection was found to be very common in PICU admissions and was associated with considerable mortality. MRSA infection is still predominantly hospital acquired in our ICU. It is particularly associated with higher complication rates and mortality than MSSA infection. Also, this organism seems to affect children with chronic diseases and disabilities more. Further, no case of vancomycin resistance was encountered in our study.
